# Cultural proficiency starts here: Drawing on staff and student perceptions to Indigenise curricula

**DOI:** 10.1002/hpja.643

**Published:** 2022-08-07

**Authors:** Shane McIver, Berni Murphy, Lizzie Curran, Ange Parrish

**Affiliations:** ^1^ Centre for Research in Assessment and Digital Learning (CRADLE) Deakin University Burwood Australia; ^2^ Faculty of Health, School of Health and Social Development Deakin University Burwood Australia

**Keywords:** Aboriginal and Torres Strait Islander, competencies, cultural capabilities, first peoples, health educator, health professional students, higher education, Indigenous curriculum

## Abstract

**Issue Addressed:**

This study reports outcomes from initial steps taken to promote cultural proficiency among our next generation, nonclinical health workforce, including addressing any tendencies towards stereotyping, biases and discrimination, when redeveloping and Indigenising curricula.

**Methods:**

This qualitative study involved purposive sampling of undergraduate students enrolled in a final‐year health promotion unit (subject), as well as staff within the Faculty of Health. Thematic analysis was applied to two datasets: (i) outcomes from a staff survey examining insights and reactions to delivering Indigenous content (n = 16) and (ii) a collection of online student posts describing their observations and reflections during delivery of an Indigenous health module (n = 91).

**Results:**

Staff survey data highlighted the need for targeted professional development and support. Findings from student data emphasised the need to embed course content that has the capacity to (i) help students understand health within an equity, social justice and human rights context and (ii) encourage students to self‐identify any conscious and unconscious biases that work against these principles in the workplace.

**Conclusions:**

This study demonstrates the usefulness of conducting a preliminary analysis prior to initiating changes to curriculum design and delivery. Strategies were identified to provide and implement renewed initiatives and directions for professional development for staff. Similarly, there was a clear need to effectively train students in cultural awareness, sensitivity and knowledges through specific and targeted resources and support throughout the span of the course.

**So What:**

Lack of knowledge and confidence among staff can negatively impact content and delivery, and ultimately, student learning outcomes. However, this was balanced by an appetite for collaboration and guidance. Findings contribute to current discourses exploring effective approaches to Indigenising discrete unit and course‐wide curricula and provide a useful template for others seeking evidence‐based approaches and ideas when aiming to improve cultural proficiency.

## 1 INTRODUCTION

The National Aboriginal and Torres Strait Islander Health Plan 2021‐2031 defines new strengths‐based and human rights approaches to wellbeing informed by cultural and social determinants of health.^1^ Produced by government in partnership with Indigenous stakeholders, the plan defines what is now expected from the health sector to “ensure mainstream services address racism and provide culturally safe and responsive care” (p. 3). For health promotion practitioners, the plan states that “health promotion activities must also understand the social and historical context of colonisation, systemic racism and intergenerational trauma” (p. 33).

Accordingly, for health promotion educators, this suggests challenges involving designing and implementing a curriculum with the capacity to develop capability among our next generation, nonclinical health workforce in a way that promotes cultural awareness in the workplace. This includes “the ability to critically reflect on one's own culture and professional paradigms in order to understand its cultural limitations and effect positive change”^2^ (p. 3). Creating strategies for responding effectively to the challenges of Indigenising curricula is timely, given the increased emphasis on graduate outcomes incorporating Indigenous cultural competency in Australian higher education, and with universities also now seeking to decolonise curriculum accordingly.^3^ The central question now concerns identifying the best way to implement changes, since graduate outcomes are seldom measured or evaluated.^4^

Among responsive pedagogies promoting Indigenised approaches to education, The National Best Practice Framework for Indigenous Cultural Competency in Australian Universities^5^ discusses Well's^6^ stage model whereby six steps define the transition from identifying unconscious cultural biases through to developing cultural proficiency. These range in increasing importance from (i) cultural incompetence, (ii) cultural knowledge, (iii) cultural awareness, (iv) cultural sensitivity, (v) cultural competence and ultimately (vi) cultural proficiency. Quoting Wells,^6^ the Best‐Practice Framework^5^ states, “at the organisational level, cultural proficiency is an extension of cultural competence into the organisational culture. For the individual and the institution, it is mastery of the [five preceding] phases of cultural competence development” (p. 59).

Drawing on Well's^6^ model to Indigenise curriculum is a useful approach, as identified by other scholars,^7^ since it is possible to recognise at what stage teaching and learning might be stuck, and how staff and students can then be moved further along the continuum towards cultural proficiency. For health promotion, this translates as a journey from “a lack of knowledge of the cultural implications of health behaviour,” through to “the routine application of culturally appropriate health care interventions and practices” associated with cultural competence.^6^(p. 192) Ultimately, proficiency involves pro‐actively seeking to refine practice though cross‐cultural engagement and ongoing professional development.^8^

Similarly, the Aboriginal and Torres Strait Islander Health Curriculum Framework (HCF)^9^ provides useful resources and guidelines, with helpful recommendations such as the “need to acknowledge cultural diversity and local context when implementing curriculum” and emphasises “the importance of educators avoiding blaming and shaming approaches.” It also assists with “development of graduate capabilities and learning outcomes with reference to a number of criteria categorised under skills, attributes, knowledge and understanding” (p. 1–11). Underpinned by eight principles, it defines a graduate cultural capability model, with each of the five interconnected cultural capabilities featuring their own set of key descriptors articulating the required “attitudes, values, skills and knowledge that students need to demonstrate to develop the associated capability” (p. 2–8). For those who find creating sustainable and authentic assessment challenging,^10^, ^11^ it also provides suggested methods of assessment as another valuable reference.

A recent systematic review examined the impact of Indigenous health care curriculum on health professional learners in preparation to deliver equitable health care.^12^ It found further understanding of educator preparedness is required, as well as the need to further explore how health professional graduates are prepared to work in Indigenous health. One main message was that opportunities exist to learn more about Indigenous health teaching and learning across learning domains (p. 525). To this end, staff surveys have often been used to examine the practices and attitudes towards teaching Indigenous content in health professional programs.^13^

Student surveys have also been used to study learner reactions and perceptions when engaging with Indigenous content. One recent study reviewed student surveys from a sample of 22 first‐year undergraduate nutrition students to identify whether integrating the Health Curriculum's Framework within the curriculum would result in increased self‐rated scores for cultural capability.^14^ Despite the statistically significant increases using the Cultural Capability Measurement Tool (CCMT),^15^ as a quantitative study it was difficult to determine the exact factors responsible for improved scores. The authors recommended both “refining of methods for evaluating changes in student cultural capabilities” and the inclusion of “qualitative research methods to understand students' perspectives and experiences of Aboriginal health curriculum” (p. 336, 337).

Online student satisfaction surveys pertaining to Indigenous content and delivery have also been used by other scholars to analyse feedback.^16^, ^17^ However, it is of interest to examine student perceptions in the absence of predetermined questions, where students are afforded the freedom to express insights and observations that arise spontaneously from their engagement throughout their studies.

As such, given the current focus on Indigenising curricula and identifying what works best, the aim of the present study was to examine how staff currently approach designing and delivering Indigenous content, and in addition, explore how students engage with content and delivery when presented with contemporary Indigenous health topics. It was anticipated findings would (i) clarify staff practices and perceptions, (ii) reveal where students were positioned along the continuum of Well's^6^ stage model and as a result, (iii) inform any necessary changes or recommendations that could contribute to building cultural proficiency for students transitioning to workplace practice.

### 1.1 Procedure

For staff, an email was circulated explaining the study, including the plain language statement and an invitation to participate in a 15‐item online survey. All submissions were anonymous. Some survey questions were similar to those posed by Wolfe and colleagues,^13^ “to identify whether Indigenous content was included in the curricula staff taught, whether they felt confident and capable of delivering curricula related to Indigenous issues, and what challenges they found in including Indigenous content” (p. 649). Examples included, “how do you typically decide what Indigenous content to teach?” and “what resources do you access for your teaching?”

Undergraduate students were recruited from a final year health promotion unit (sometimes referred to as a subject) that examined contemporary health issues, where media reporting was compared to academic, evidence‐base findings. A core unit within the public health and health promotion course, it draws on a pedagogy of discomfort as described by Boler,^18^ and therefore one of the unit's main objectives was to encourage students to reflect on their own values, attitudes and beliefs as they examined different controversial health topics each week. As an example, content relevant to this research included case studies, images, information and statistics that challenged stereotypes and clichéd representations of Indigenous health delivered in a singular module over 1 week, as well as examination of racism and discrimination and their impacts on health outcomes.^18^, ^19^ Content was presented through seminars via on‐campus and online formats, supported by online references and resources.

To capture their observations, students were required to reflect on the Indigenous health module's content and delivery, including readings and write “blogs” on the online discussion forums relevant to the topic.^20^ Although their writing ultimately served to inform a broader reflective practice assessment task, the actual blogs were not graded. To promote high standards of critical thinking and writing, resources were provided to help students understand reflective practice as well as the blogging process.^21^, ^22^, ^23^ The research study was explained in the last week of the trimester once all assessment tasks were submitted. Students were informed if they chose to participate, their assessments, as well as their discussion forum blogs, were all considered to be data. For the purposes of this study's aims, analysing student blogs relevant to Indigenous health was of most interest, given they provided noncensored and authentic accounts of their initial insights, reactions and reflections.

After receiving their final grades and feedback, students were invited to log on to the unit's site and access the online link (via RedCAP) to the plain language statement and digitally sign the consent form. To minimise potential coercion, students were also informed teaching staff would not be aware of who took part, that all submissions would be de‐identified by an independent research assistant prior to analysis, and any requests to withdraw would be actioned. It was anticipated analysing staff and student perspectives would serve as a useful springboard for examining current teaching practices as well as student observations and insights, and therefore inform targeted efforts to Indigenise the curriculum effectively.

## 2 METHODS

### 2.1 Design

This study used a descriptive qualitative research design^24^ informed by (i) outcomes from a staff survey and also (ii) reviewing a collection of online student posts that students eventually incorporated into one of their assignment tasks. Notably, the straightforward nature of descriptive qualitative research is particularly useful before developing an intervention.^25^ An inductive analytic approach was then applied to both datasets using thematic analysis as defined by a framework method^26^ described below.

### 2.2 Sample

Participants included teaching staff across a single school within the Faculty of Health, as well as a purposive sample of undergraduate students enrolled in a singular health promotion unit in their third and final year. Recruitment commenced once university ethics committee approval was obtained for staff and students alike (DUHREC 184 and HEAG‐H 154 respectively).

Among the 16 staff who completed the survey, only one staff member identified as Indigenous. Most participants taught three units per year (range 1‐5), and although many also included some content relating to Indigenous health (81%), some did not (19%). Students providing informed consent (N = 386; n = 91) included a combination of on‐campus (n = 11) and off‐campus (n = 80) students, with ages ranging from 20 to 57 across the cohort. The average age ranged between 25 and 27 years old.

### 2.3 Analysis

The framework method^27^ was an appropriate fit for the multi‐coder data analysis for several reasons.^26^ It supports a thematic analysis and mapping process and is suited to studies involving a multidisciplinary research team. In the case of the present study, this included a codesign approach involving collaborations among a mix of Indigenous and non‐Indigenous research colleagues, some of whom have worked on curriculum development before and others who have not. The framework method accommodates a range of skillsets and can be considered an inclusive approach, since it promotes equality rather than research team hierarchies as a process, and does not discriminate between those with minimal or extensive analytic experience.^26^ Guidance from a knowledgeable qualitative researcher is strongly recommended, however, and was implemented within this study.

The framework method inherently embeds practices to augment rigour, such as providing transparent audit trails and links within the data, countering some of shortfalls to which some forms of thematic analysis are prone.^28^ It involves a series of stages, including (i) transcription, (ii) data immersion, (iii) coding, (iv) developing an analytic framework, (v) applying the framework, (vi) charting the data within a matrix and (vii) interpreting the data. Examples of detailed approaches to each of these steps can be found in further references.^26^, ^29^, ^30^

Given qualitative research's focus on obtaining rich and robust data,^31^, ^32^ indicated by factors such as saturation^33^ and information power,^34^ the total dataset for both cohorts was considered sufficient to meet these criteria and yield useful findings. The overview of key themes and relevant detail are presented in the section below.

## 3 RESULTS

Main themes for the staff cohort were as follows:

### 3.1 Knowing where to start

For the question, “Do you include Indigenous content in all your units?,” 37% reported “yes,” 44% stated “some” (a total of 81%) and 19% reported they did not. Therefore, a consistent approach was typically not present. When considering what approach to adopt and what information to include in the units they teach, many commented there were difficulties regarding where to begin, with the default approach often skewed towards offering content representing a deficit model of Indigenous health and problematising related topics. This could be a flow‐on from the ways Indigenous health is often reported generally. One participant observed, “I always try … to move beyond the ‘deficit’ way of thinking. However, this can be challenging … as it is common in government reports to present Indigenous statistics in comparison to nonindigenous data” (#3).

Knowing what content would be useful required reflection on how to position it within a meaningful context and what to offer, and often depended on the individual staff member, given there were no established support systems or guidelines to promote a consistent approach. Although some staff consulted with colleagues, they still struggled to know what would be most appropriate. Despite this, there was an appetite to know more. As one participant observed, “I would be keen to know how else I can incorporate Indigenous content into these units” (#7). Notably, each staff member described a different approach to content development, with no apparent similarities or reference to any particular guiding principles.

### 3.2 Feeling capable versus feeling unsure

When teaching Indigenous content in general, half reported feeling capable (50%), which increased when teaching one's own general discipline content to Indigenous students (63%). However, when teaching Indigenous topics to Indigenous students, the number of staff feeling capable dropped (44%), with some feeling unsure of their abilities (25%), or even feeling awkward (19%).

### 3.3 Feeling the fear and doing it anyway

Among those who felt awkward or unsure, staff were aware of their own knowledge gaps that gave rise to uncertainty, but there was also uncertainty about how to resolve that situation. As one participant wrote, “I am less certain about discussing case studies and impact on communities” (#9), and “I do not know, what I do not know! … so sometimes I am unsure about acceptability of my approach” (#1). There was also a consensus that although staff might not always get it right, or that they might offend or cross a line, that something was better than nothing. Many expressed that preparation is key, possibly providing an antidote to the anxieties that often arise in its absence.

### 3.4 Expert versus facilitator

Participants noted that positioning oneself as being the expert forces assumptions around being the keeper of power and knowledge, adding unnecessary pressure, whereas being a facilitator can create space for opening questioning and investigating together, encouraging collaboration and information sharing. There is an inherent willingness to learn for staff and students alike. Some reported how the position of expert can also promote a sense of imposter syndrome, creating further inner tension: “I do not feel I understand enough about how Indigenous students would feel about a nonindigenous person teaching about their culture, beliefs etc. I would worry I would not do such an amazing culture, and a very significant history within Australia, justice” (#7).

### 3.5 Knowing where to look

Most participants drew on journal articles as resources, followed by videos, other readings and interviews as external teaching tools. Many expressed interest in collaboration with colleagues, training and guidance. Specifically, this related to a desire for professional development and collaborations with Indigenous academics, plus content from Indigenous people and curated sources. Comments included: “I would find talking with someone to support me to incorporate more Indigenous content into my units helpful. I think I would then feel more comfortable talking about it without worrying I am going to unintentionally offend someone” (#7), and “It's more than resources. It's deep training and guidance. It's having an Indigenous mentor to guide and show the way, feel safe to ask questions” (#1).

In many cases, participants were frustrated with the challenges represented by not knowing who to contact, including how to partner with Indigenous individuals, how to locate contemporary information, and being able to have more time for preparing content, along with feelings of being pressured. Not all participants were enamoured with current shifts in developing a reflexive focus on teaching and learning relevant to Indigenous content. For example, one wrote, “… now we are getting to this point where there are ‘rules’ around who can do the content and so forth. I understand and appreciate those, but they don't sit with how education works. If it gets too hard, then I just won't include this content” (#15) and “…‘Do we’ or ‘Don't we’ and ‘Should we’ or ‘Shouldn't we’. There is no consistency on this and sometimes staff are too opinionated on their attitudes around this and bring in the ‘fear’ to teach it. Sometimes indigenous educators also make us feel that we shouldn't teach it even if we would like to” (#5).

Although most (68%) had participated in some form of professional development, there were some who had not (32%). Overall, there was a strong desire among the cohort to understand more about Indigenous ways of knowing and explore complementary methods of teaching and learning.

### 3.6 Student outcomes

Thematic analysis of student data produced the following:

### 3.7 Shock and awe

Students were blindsided by discovering the Indigenous stories of inequities driving reported health outcomes statistics, with many stating how the process was “eye‐opening.” As one student wrote, “I feel absolutely disgusted, disappointed, angry and upset. To be honest growing up I had no idea about any of these issues, I would just listen to what I had heard on the news or from family” (#20). Increased awareness gave rise to a sense of shock and surprise. One student commented, “It shocks me how little aware I personally am, of something that should be basically common sense. The hardships these people faced in the past and how that has affected them to this day, not just physically but emotionally is heartbreaking” (#31).

Students also began recognising and discussing white privilege, with some beginning to identify their own role in the process: “really made me think about how I perpetrate racism and how I can better respond when I am called out for it – for example, saying thank you rather than getting defensive” (#41). Surprise at the current realities of Indigenous health was accompanied by a second theme, described below, where students began to realise the limited nature of what they believed they already knew.

### 3.8 Thinking we know (and realising we do not)

Students were under the impression familiarity with statistics equated to familiarity with the determinants of Indigenous health generally. However, their own deeper inquiry helped them realise not only was their understanding poor, but in some cases, there was recognition of internalised negative stereotyping that was previously un‐identified. One student commented, “…before the seminar I wouldn't have considered myself as uneducated about discrimination and racism as I now think I am?” (#23). Such self‐realisations and self‐disclosure were common throughout the data.

Although there was greater awareness regarding the mechanisms driving disadvantage and negative health outcomes for Indigenous individuals and communities, students appeared to maintain their own perspectives as central throughout their observations. Notably, they were aware of this trend, and questioning it as well. As one student wrote, “although I have previously been aware of the inequities and disparities Indigenous people are constantly facing, I may have thought of these issues in which Indigenous people face at more of a surface level view and from an outsider's perspective” (#34).

### 3.9 Uncertainty about how to process personal powerlessness

Students reported how they were not only unsure how to process their own feelings about seeing injustice as a bystander and not speaking up, but also began to recognise the lived experience of what injustice means and how it impacts health for others. One participant wrote, “I found these topics to be quite confronting and triggered feelings of anger and sadness for these vulnerable population groups. I was previously aware of the gap that Indigenous people experience and also the effects of racism, but I did not realise just how prevalent these health issues currently are in Australia” (#28). There was distress and frustration at human rights abuses and the failure to address them, but there was also an uncertainty about how to initiate conversations or actions that help to heal the rifts: “this topic was frustrating to me as I can't understand how we can treat other humans this disgracefully and get away with it” (#36).

### 3.10 Speaking truth to power

Students recognised the difficulties associated with being heard. Many identified the trauma associated with explaining and describing disrespect and abuse, yet it seemed as if this was the only pathway for Indigenous people to be heard and for change to occur, perpetuating the trauma and translating as a completely unfair process. Many commented about how filtered and limited information has been the case for years, such as only hearing about colonisation from the white perspective, and that this is how they were taught in schools. Some mentioned how their early education tended to sugar‐coat history by delivering dreamtime stories rather than helping students learn about what truly happened historically and what needs to change. One student reported, “I feel like I barely know about the history of the first Australians and everything that I have learnt has come from the point of view of those who settled in Australia. I think it's important that we have more of an education about the history and exactly what happened” (#18).

### 3.11 The call for accountability

With increased awareness came increased outrage at the inequities associated with Indigenous health, demonstrating deeper understanding of the systemic mechanisms responsible for the outcomes. One student stated, “Overall it is a bigger political issue, stemming from bad politics, poor social policies and programs and unfair economic arrangements, leading to fewer opportunities” (#15). Typically, comments reflected anger and frustration that the government is expected to do more, society is racist (with some students self‐identifying with those values and subsequently questioning them), “others” should do more, and ‘others’ aren't doing enough or not fast enough. For example: “Why aren't we getting this right? I became increasingly angry and frustrated that over the decades of reporting, consultations and papers we've commissioned, we've seemingly implemented none of these learnings?” (#26).

Self‐blame was also a common reaction: “I was shockingly ignorant to the inhumane and racist actions of our governments and sequential leaders before them, who have blatantly neglected actionable change” (#12). There was also recognition of some of the changes required for moving forward: “What we can learn from Indigenous people is a really important point… with this comes a rise in status and sense of purpose within the community which has largely been torn down over 200 years,” and “maybe it's time for us to stop talking and start listening!” (#42).

### 3.12 Becoming an agent of change

Some students felt inspired and compelled to learn more and work in this field. They expressed wanting to be an agent of change but cited not knowing how to do that or what's really required as the main barriers. There was a desire to want to learn how to stand up to racism and not be oblivious or be a bystander. This included recognition of needing to listen more and develop the skills to engage in respectful dialogue. These intentions were reflected in comments such as, “We must educate more about these issues, especially children and teenagers … I hope that I can learn more about these issues and gain knowledge around health equality so that I can educate other people. I am hoping that one day I will have a chance to make a change in the world” (#33). Another wrote, “I will now strive to seek as much information as I can on the current health state of Aboriginal and Torres Strait Islanders to help educate and inform myself for future growth that I can also apply as a practitioner” (#45).

As an overall observation, throughout the student dataset analysis, a noticeable tendency to use a language of separation with terms such as “these people,” “other,” “me versus less fortunate,” was prevalent, and should be mentioned. Implications from this and other findings are reviewed in the discussion section below.

## 4 DISCUSSION

Outcomes from this study aligned with many of the HCF's^9^ guiding principles, graduate capabilities and their descriptors. For example, there was a deepening appreciation among students that the learning process itself was as important as the information within course content. Students could also see how reflexivity is integral to respectful professional practice. For staff, data emphasised the necessity for ongoing professional development and support (p. 31). Recognising the role of racism and white privilege arose through reflection, as per the HCF's graduate capabilities, and these insights were identified by the students themselves as part of their increased awareness. Advocacy, another HCF graduate capability, was expressed through desire to be an agent of change and take an active role to support equitable health.

Data from the staff survey afforded comparisons with previous research that had investigated similar survey questions, such as Wolfe et al,^13^ where more than half the number of staff included Indigenous content (63% and 81% for the present study, respectively). Both studies also identified there were staff who did not include any such content (43% and 19%, respectively) as well. Both studies also found many staff felt less capable or even awkward when teaching Indigenous‐related content to Indigenous students, in comparison to teaching general, discipline‐specific content. Wolfe et al^13^ reported this is also consistent with other research.^35^, ^36^ Despite these tendencies, staff participants from both Wolfe et al's^13^ cohort and this study expressed interest in training and being able to source guidance for content redesign. Although Wolfe et al's^13^ group had participated in professional development at a higher percentage (90%) than those in the present study (68%), both studies found staff actively seek and would appreciate further opportunities to build on these foundations. This is not always the case, however, as reported in other research where staff have resisted change.^37^, ^38^

It is natural to expect that undergraduates who are about to enter the workforce would already be aware of the realities behind the government reports and statistics, yet data revealed students had multiple knowledge gaps and lack of experiential understanding. Indeed, many students initially identified with the first step of Well's^6^ stage model, harbouring unconscious biases. Student data revealed that reflection, increasing self‐awareness, and questioning personal values and beliefs, triggered shifts into the subsequent stages of developing cultural awareness and knowledge, and that this approach to learning might be necessary for such progress to occur. These positive changes could well be attributed to the use of Boler's^18^ pedagogy of discomfort which was embedded throughout the unit from which students were recruited, since the ways it challenges assumptions and integrates reflexivity can promote radical shifts in critical thinking.^39^ As such, educators might consider embedding it within units to promote and augment reflective practice and self‐awareness.

Data from both cohorts illustrate how the consequences of shortcomings in Indigenous health content and delivery could seemingly translate into a culture of information‐disconnect, with minimal critical inquiry or reflexivity from staff and students alike. Educators seeking to Indigenise curricula will benefit from examining staff and student perspectives through their own data collection and analyses, since it could increase awareness about how staff decision‐making impacts student learning in ways that might otherwise go unnoticed.

Essentially, outcomes from the present study suggest three key, overarching considerations for effectively initiating an Indigenous curriculum: (i) embedding the above points and mapping Indigenous content across course curricula, (ii) the importance of scaffolding and building relevant content and delivery to address any limited understanding among students and (iii) to use the opportunity to consult with Indigenous stakeholders who can advise accordingly through ongoing professional training and support. Naturally, the HCF^9^ also provides useful guidance, with its categories of novice, intermediate and entry to practice corresponding to first, second and third year, as well as information and expectations relating to each, assisting both vertical and horizontal mapping within a complete course.

In this case, several considerations and changes that could be made at individual unit and course levels to improve graduate outcomes became apparent after further consideration of the findings through the lens of the HCF and Best‐Practice frameworks. For example, in the present study where there is a focus on public health and health promotion, several strategies for change that staff can implement were identified based on outcomes from both staff and student datasets. These included, but were not limited to:

1. Embed reflexive approaches to teaching as a starting point (eg, examining underlying assumptions of self, others and context),^40^ since this assisted students to self‐identify their own values and beliefs.
2. The need to unpack key government initiatives (eg, Closing the Gap), statistics and targets, aspirations, underlying causes driving inequities and inequalities, and social determinants from Indigenous perspectives.^41^ Especially since findings indicated students were often unaware of the real‐world, lived experience for individuals and communities relevant to Indigenous health outcomes.
3. The need to deconstruct the mechanisms and impacts of individual racism, systemic racism, overt and covert racism and how these influence health.^19^
4. Given learning involves more than content alone,^10^ to include assessments and/or modules in several units and incorporate Indigenous health in a coordinated and scaffolded way to build awareness, knowledge, skills and capabilities. It is useful if these feature self‐evaluative methods in alignment with integrative and sustainable assessment.^42^
5. Staff feedback emphasised the importance of identifying resources that provide an antidote to deficit models of Indigenous health. Examples include more narratives, case studies and programs that demonstrate inspirational examples of codesign and empowerment.^3^, ^43^
6. For personal and professional development, students need to be taught how to manage uncomfortable emotions and reflections, how to process them and arrive at a place that allows them to move forward. Given the tendency towards “othering” which became apparent when analysing student data, this would also involve guidance on language, such as how to hold culturally appropriate conversations and also how to respectfully challenge opinions as part of inclusive practitioner goals.^44^, ^45^

### 4.1 Limitations

Staff and students self‐selected, and therefore it is not known if those who did not participate had different views. However, themes from the dataset provided useful insights to guide initial steps for appropriate and necessary curriculum redevelopment. In addition, findings from this study concentrated on the perspectives from two specific cohorts, and therefore cannot be generalised. However, data from the staff survey validates some trends also identified by Wolfe et al,^13^ thereby suggesting there might be common patterns across institutions. Indeed, it would be interesting to participate in cross‐institutional research in this regard, to further broaden collaborative approaches and knowledge‐sharing.^46^ Also, this study was not Indigenous‐led, and therefore it should be acknowledged this would need to be rectified in any subsequent studies of this nature. All these factors point to opportunities for future research, as educators begin to discover what works for their own purposes, and how recommendations arising from examining staff and student experiences are subsequently developed, implemented and evaluated over time.

## 5 CONCLUSION

This study conducted an analysis of staff and student perspectives to identify best practice when Indigenising curricula for a specific public health and health promotion course. It examined how staff currently approach designing and delivering Indigenous content, and in addition, explored how students engaged with content and delivery when presented with contemporary Indigenous health topics within a single unit. Findings demonstrated that being aware of staff and student observations and insights can be a valuable first step in what essentially becomes a collaborative work in progress to inform curriculum redevelopment, and to avoid haphazard approaches.

Staff welcomed ongoing, iterative training to build knowledge, skills and confidence over time. Guidance was also sought for appropriate content redesign in cases where staff felt hesitant and uncertain when teaching information relating to Indigenous health. Through increased awareness and reflexivity, students were academically challenged, began to question their certitude, and were willing to examine their own biases in the process. As such, they readily identified their limited knowledge and actively began seeking understanding and skills more aligned with cultural awareness, knowledge and sensitivity, without being instructed to do so. Accordingly, this study provides a template for others seeking to conduct their own investigations to inform curriculum redevelopment suitable to their own requirements.

Future research could draw on these preliminary results as efforts to Indigenise curriculum develop. Cross‐institutional collaborations and knowledge‐sharing are encouraged, since this is shared work with many higher education institutions who are increasingly becoming heavily invested in the outcomes.

## CONFLICT OF INTEREST

The authors declare no conflict of interest.
